# Let-7b Regulates Myoblast Proliferation by Inhibiting *IGF2BP3* Expression in Dwarf and Normal Chicken

**DOI:** 10.3389/fphys.2017.00477

**Published:** 2017-07-07

**Authors:** Shumao Lin, Wen Luo, Yaqiong Ye, Endashaw J. Bekele, Qinghua Nie, Yugu Li, Xiquan Zhang

**Affiliations:** ^1^Department of Animal Genetics, Breeding and Reproduction, College of Animal Science, South China Agricultural UniversityGuangzhou, China; ^2^Guangdong Provincial Key Lab of Agro-Animal Genomics and Molecular Breeding, and Key Lab of Chicken Genetics, Breeding and Reproduction, Ministry of Agriculture, South China Agricultural UniversityGuangzhou, China; ^3^School of Life Science and Engineering, Foshan UniversityFoshan, China; ^4^College of Veterinary Medicine, South China Agricultural UniversityGuangzhou, China

**Keywords:** let-7b, IGF2BP3, chicken, myoblast proliferation, dwarf

## Abstract

The sex-linked dwarf chicken is caused by the mutation of growth hormone receptor (*GHR*) gene and characterized by shorter shanks, lower body weight, smaller muscle fiber diameter and fewer muscle fiber number. However, the precise regulatory pathways that lead to the inhibition of skeletal muscle growth in dwarf chickens still remain unclear. Here we found a let-7b mediated pathway might play important role in the regulation of dwarf chicken skeletal muscle growth. Let-7b has higher expression in the skeletal muscle of dwarf chicken than in normal chicken, and the expression of insulin-like growth factor 2 mRNA binding protein 3 (*IGF2BP3*), which is a translational activator of IGF2, showed opposite expression trend to let-7b. *In vitro* cellular assays validated that let-7b directly inhibits *IGF2BP3* expression through binding to its 3′UTR region, and the protein level but not mRNA level of *IGF2* would be reduced in let-7b overexpressed chicken myoblast. Let-7b can inhibit cell proliferation and induce cell cycle arrest in chicken myoblast through let-7b-IGF2BP3-IGF2 signaling pathway. Additionally, let-7b can also regulate skeletal muscle growth through let-7b-GHR-GHR downstream genes pathway, but this pathway is non-existent in dwarf chicken because of the deletion mutation of *GHR* 3′UTR. Notably, as the loss binding site of *GHR* for let-7b, let-7b has enhanced its binding and inhibition on *IGF2BP3* in dwarf myoblast, suggesting that the miRNA can balance its inhibiting effect through dynamic regulate its binding to target genes. Collectively, these results not only indicate that let-7b can inhibit skeletal muscle growth through let-7b-IGF2BP3-IGF2 signaling pathway, but also show that let-7b regulates myoblast proliferation by inhibiting *IGF2BP3* expression in dwarf and normal chickens.

## Introduction

MicroRNAs (miRNAs) are a class of small conserved noncoding RNA that negatively regulate target gene expression through complementarily binding to the 3′ untranslated region (3′UTR) of mRNAs. Recent studies indicate that miRNAs are involved in the regulation of skeletal muscle development (Luo et al., 2013). The let-7 miRNA family is conserved across diverse animals, functions to control late temporal transitions during development (Grosshans et al., 2005). During the last decade, the involvement of let-7 in regulating cell differentiation has been analyzed in various contexts, including neural cell specification, stem cell maintenance and hematopoietic progenitor differentiation (Wulczyn et al., 2007; Oshima et al., 2016; Peng et al., 2017). However, its roles in skeletal muscle development still remain unclear. Let-7b, a member of the let-7 miRNA family, has been found to inhibit chicken growth by repressing *growth hormone receptor (GHR)* gene expression (Lin et al., 2012). It can also regulate fat synthesis and cell proliferation through *GHR*-mediated signaling pathways (Lin et al., 2012). Additionally, our previous study indicated that let-7b might be involved in the regulation of skeletal muscle development (Luo et al., 2016). But its precise roles and regulatory pathways in skeletal muscle still remain to be explored.

Insulin-like growth factor 2 mRNA-binding protein 3 (IGF2BP3) is one of an important members for insulin like growth factor mRNA binding protein (IGFBP) family, which has growth promoting effects during cell developmental processes (Nielsen et al., 1999, 2001, 2002; Bell et al., 2012). Previous studies indicated that IGF2BP3 is a secreted protein that can bind to insulin like growth factor 2 (IGF2) and function as carrier protein in the circulation (Bell et al., 2012). IGF2BP3 can also regulate IGF2 localization and play an important role in cell proliferation and migration (Jones and Clemmons, 1995; Nielsen et al., 2002; Bell et al., 2012). It was found that IGF2BPs participate in the physiological regulation of IGF2 production (Nielsen et al., 1999). IGF2 is a master switch governing the initiation of skeletal muscle development (Ge et al., 2011), and it perhaps function as an autocrine or paracrine factor in stimulating both proliferation and differentiation of muscle cells (Florini et al., 1991). Therefore, IGF2BP3-mediated regulation of IGF2 function may play roles in skeletal muscle development.

The body weight, muscle fiber diameter and muscle fiber number would be reduced in sex-linked dwarf chicken (Knizetova, 1993; Luo et al., 2016), but the precise regulatory pathway involved in the dwarf chicken skeletal muscle growth still remain unclear. Our previous results have showed that the expression of let-7b was up-regulated in the skeletal muscle of dwarf chicken, and its expression showed opposite expression trends to the mRNA expression of *IGF2BP3* (Lin et al., 2012). To further understand the relationship between let-7b and *IGF2BP3*, and investigate the regulatory roles of let-7b in skeletal muscle development of dwarf and normal chickens, we use primary myoblast from dwarf and normal chickens to analysis let-7b mediated regulation of *IGF2BP3* expression and muscle cell proliferation *in vitro*. The findings of this study would be beneficial to understand the function and regulation of let-7b in skeletal muscle development of the dwarf and normal chickens.

## Materials and methods

### Ethics statement

All experimental protocols were approved by the South China Agricultural University Institutional Animal Care and Use Committee. And the methods were carried out in accordance with the regulations and guidelines established by this committee. Animal experiments were carried out in compliance with animal care protocols and all efforts were made to minimize suffering.

### Animals

The chickens used in this study were consistent with our previous study (Lin et al., 2012). In short, the central muscle of the gastrocnemius was separated from 9 female dwarf chickens and 9 female normal chickens. Three pooled RNAs with each pool containing RNA from 3 muscle samples were used for mRNA and miRNA expression analysis. For protein expression analysis, we collected another 4 chickens with the same breed, age, sex and muscle collection site as those used in mRNA and miRNA expression analysis.

### Cell culture

Chicken primary myoblasts were isolated from the leg muscle of 6 dwarf or 6 normal White Recessive Rock chickens at E11. The isolated leg muscles were minced and pooled in growth medium (GM) consisting of RPMI-1640 medium (Gibco), 20% fetal bovine serum (Hyclone), 10% chicken embryo extract and 0.2% penicillin/streptomycin (Invitrogen). To release single cells, the suspension was shaken by repetitive vortexing and filtered to remove large debris. The cells were then collected by centrifugation at 350 g and resuspended in GM. Serial plating was performed to enrich myoblasts and eliminate fibroblasts. Chicken embryo fibroblast cell line (DF-1) was cultured in high-glucose Dulbecco's modified Eagle's medium (Gibco) with 10% fetal bovine serum and 0.2% penicillin/streptomycin.

### Quantitative polymerase chain reaction (qPCR) analysis

Real-time quantitative PCR with SYBR Green was used to detect relative mRNA expression levels of the major genes in the signaling pathway. Using published genome sequences, the Primer Premier 5 software was used for primer design (Table 1). In the present study, the Ct value was applied to detect the mRNA expression of the samples, and three replicates were set for each sample. The 2^−ΔΔCt^ method was used to measure gene expression with β *-actin* as the reference gene (Kenneth and Thomas, 2001).

**Table 1:** Sequences of primers used for qRT-PCR.

**Genes**	**Sequence**	**Annealing temperature (°C)**	**Product (bp)**	**Accession No**.
*IGF2BP3*	F:5′GCTGCTGCTGCTTCATATCCAC3′	60	103	NM_001006359.1
	R:5′CCTGCTTGCCAATAATAGCTCCA3′			
*IGF-2*	F: 5′AGGATCAACCGTGGCATTGT3′	60	92	NM_001030342.1
	R: 5′TCTGACTTGACGGACTTGGC3′			
*IGF-2R*	F: 5′GAGGCTTTTGTGGACGGAGA3′	60	106	NM_204970.1
	R: 5′CTGCTCCAGGTGGGCAATTA3′			
*IGF-1*	F:5′TGGCCTGTGTTTGCTTACCTT3′	60	91	NM_001004384.2
	R:5′TACGAACTGAAGAGCATCAACCA3′			
*IGF-1R*	F:5′GTACTTCAGTGCTTCGGATGTG3′	61.1	397	NM_205032.1
	R:5′ CTTCTTCAGAGTTGGAGGTGCT3′			
β-*actin*	F:5′CCCCATGCCATCCTCCGTCTG3′	61	265	NM_205518
	R:5′CCTCGGGGCACCTGAACCTCTC3′			

### Luciferase reporter assays

Based on the data in GenBank, primers for amplifying the *IGF2BP3* 3′UTR region were designed (Table 2). The plasmid pmirGLO-IGF2BP3-3′UTR was prepared for verification of target relationship between let-7b and *IGF2BP3* mRNA. Two types of plasmids, the wild-type, and a mutant with let-7b potential binding site deleted were prepared. Let-7b mimic (50 nM) and pmirGLO-IGF2BP3-3′UTR (200 ng) were co-transfected into DF-1 cells (3 × 10^4^ cells) by using Lipofectamine 3000 reagent (Invitrogen) according to the manufacturer's instructions. After 48 h, dual-luciferase reporter assays were conducted to analyze the activities of luciferases. The luminescent signal was quantified using Synergy 2 Multi-mode Microplate Reader (Biotek) and analyzed with Gene5 software (Biotek).

**Table 2:** Sequences of primers used for vector constructions.

**Genes**	**Sequence**	**Annealing temperature (°C)**	**Product (bp)**
*IGF2BP3*	F:5′TAC***GAGCTC***AGAAGAAACACACGAGG3′	60	453
(NM_001006359.1)	R:5′CGTG***TCTAGA***TTACCCACCTTACTCCC3′		

*Sequences in bold represent the restriction site, and the underlined sequences represent the protective base for the restriction site*.

### Western blot analysis

**Figure 1:**
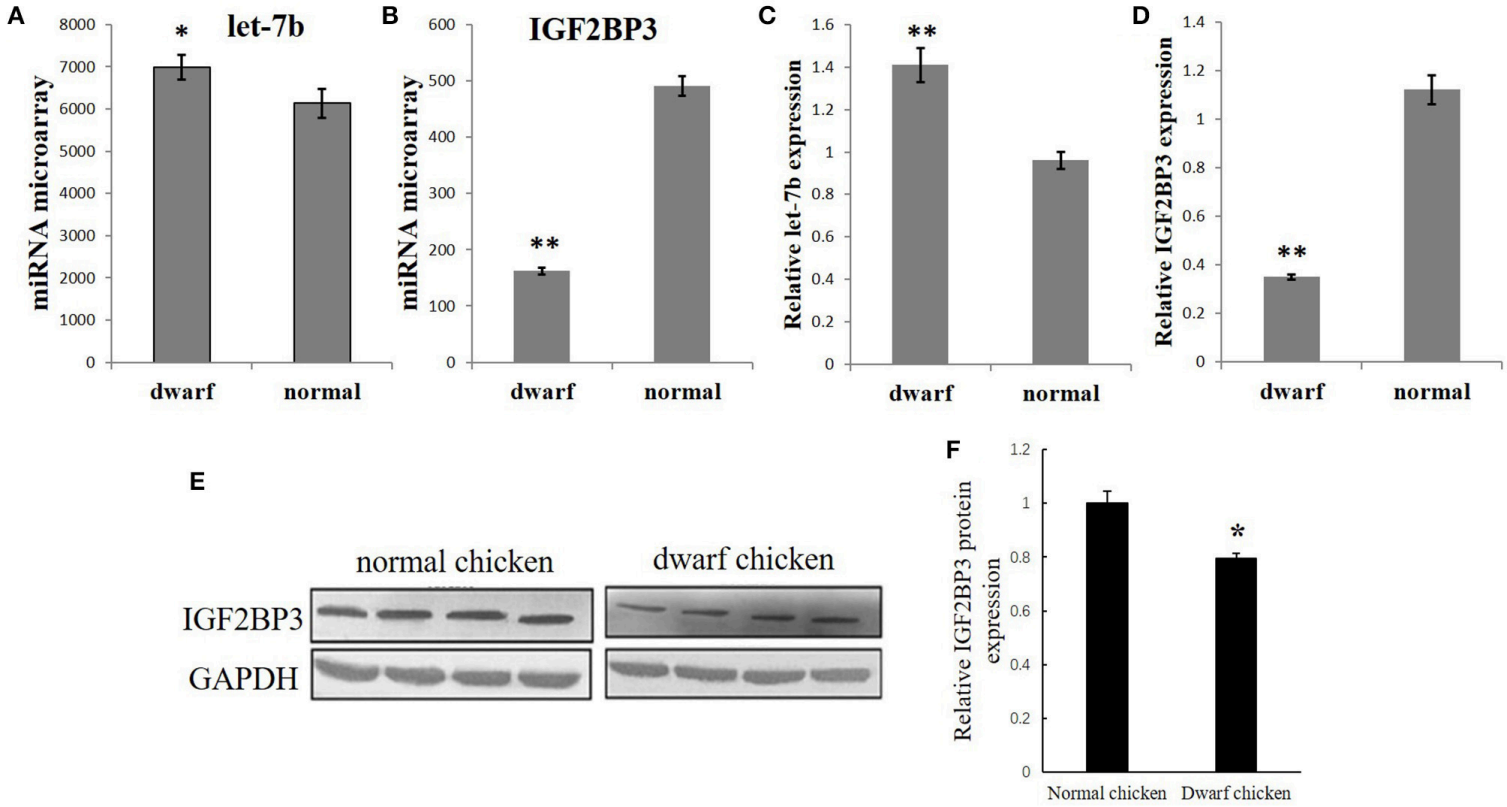
Opposite expression trends between let-7b and *IGF2BP3* in the skeletal muscle of dwarf and normal chickens. **(A)** Expression of let-7b in skeletal muscle of dwarf chicken at 7-week-old was up-regulated than normal chicken (6,992 vs. 6,137) by microarray analysis. Values are represented as mean ± S.E.M. (*n* = 9). **(B)** Expression of *IGF2BP3* mRNA in skeletal muscle at 7-week-old was down-regulated in dwarf chicken compared with the normal chicken (491.2 vs. 162.4) by microarray analysis. Values are represented as mean ± S.E.M. (*n* = 9). **(C)** qPCR validation of let-7b expression in skeletal muscle of dwarf and normal chickens. Values are represented as mean ± S.E.M. (*n* = 9). **(D)** qPCR validation of *IGF2BP3* mRNA expression in skeletal muscle of dwarf and normal chickens. Values are represented as mean ± S.E.M. (*n* = 9). **(E,F)** Compared with normal chicken, the expression of IGF2BP3 protein in dwarf chicken was down-regulated by 20% by western blot analysis. Values are represented as mean ± S.E.M. (*n* = 4). Independent sample *t*-test was used to analyze the statistical differences between groups. ^*^p < 0.05; ^**^p < 0.01.

Total protein was extracted from skeletal muscle or transfected cells which were lysed in Radio-Immunoprecipitation Assay buffer supplemented with protease and phosphatase inhibitor mixture (Sigma-Aldrich, USA), and protein concentrations of cell lysates were determined. For Western blot analysis, equal amounts of protein samples were separated by 12% SDS-PAGE and transferred onto polyvinylidene fluoride membranes (Millipore, USA). Blots were blocked using 5% skim milk, followed by incubation with primary antibody anti-IGF2BP3 (Novus Biologicals, USA, 1:500 dilution), and rabbit anti-GAPDH (Santa Cruz, 1:5,000 dilution). Immune complexes were visualized by incubation with specific secondary antibody conjugated to horseradish peroxidase (HRP, Santa Cruz) and membranes were detected with BeyoECL Plus kit (Beyotime, China). Imaging was performed with Bio-Rad imaging system, and the band gray value was analyzed via the Image J software (https://imagej.nih.gov/ij/). The protein expression were presented as the ratio between IGF2BP3 gray value and GAPDH gray value. We set the mean expression value of NC group (**Figure 3**) or normal chicken group (Figure 1) to 1, and the other group was a fold change comparing to NC group or normal chicken group.

### Enzyme-linked immunosorbent assay (ELISA)

Cell supernatants were collected at 36 h after let-7b transfection, and then the supernatant were centrifuged for 15 min at 1,000 × g. Cell debris was removed and assayed immediately. The levels of IGF2 were determined using chicken IGF2 ELISA kit (CUSABIO BIOTECH Co. Ltd. China), following the manufacturer's instructions. Briefly, polystyrene 96-well plates were treated with 100 μL of biotinylated detection antibody for 2 h at 37°C. Each well was aspirated and washed, and the above process was repeated two times for a total of three washes. After the last wash, any remaining washing buffer was removed by aspirating or decanting. The plate was inverted and blotted against clean paper towels. Then the plates were incubated with HRP- avidin for 1 h at 37°C and washed again. The signal was developed after addition of TMB Substrate for 15–30 min at 37°C (protect from light) and the reaction was stopped by adding 50 μL of Stop Solution. A microplate reader (Bio-rad, USA) was used to detect the signals at 450 nm with correction at 540 nm.

### Cell cycle analysis

After 36 h transfection of miRNA mimic or the negative control (NC) mimic, chicken primary myoblasts were collected and fixed in 75% ethanol overnight at −20°C. After ethanol fixation, the cells were stained with 50 μg/mL propidium iodide (Sigma) containing 10 μg/mL RNase A (TAKARA) and 0.2% (v/v) Triton X-100 (Sigma) for 30 min at 4°C. BD Accuri C6 flow cytometer (BD Biosciences) was subsequently used to analyze the cell cycle, and the data analysis was performed using FlowJo 7.6 software (Verity Software House).

### Immunofluorescence

Immunofluorescence assays were performed as previously described (Luo et al., 2014). The following antibody was used for immunofluorescence: anti-desmin (Bioss, China), Goat Anti-rabbit IgG/FITC antibody (Bioss, China).

### Statistical analysis

All data shown are mean ± S.E.M. with at least three samples or cultures per group and three wells per culture. Well was considered the experimental unit for cell culture applications. For **Figures 3F,J**, Duncan's Multiple Range Test was used to compare differences among mean values at 5% level of significance. For the other results, we performed statistical analysis by using independent sample *t*-test through SPSS. We considered *p* < 0.05 to be statistically significant. ^*^p < 0.05; ^**^p < 0.01.

## Results

### Opposite expression trends between let-7b and IGF2BP3 in the skeletal muscle of dwarf and normal chickens

**Figure 2:**
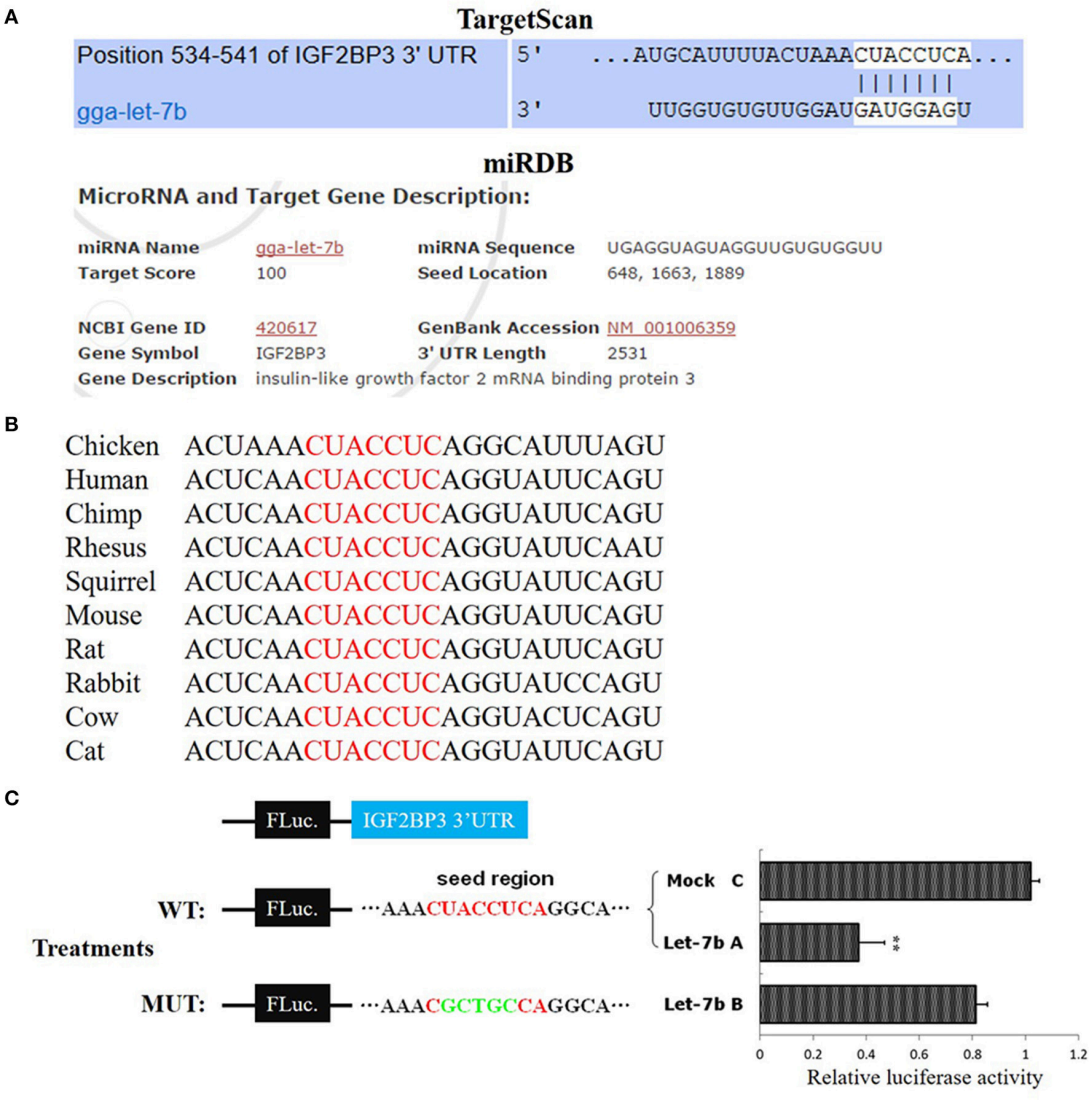
*IGF2BP3* is a target gene of let-7b. **(A)** The potential binding site of let-7b in the chicken *IGF2BP3* mRNA 3′UTR predicted by TargetScan and miRDB. **(B)** The potential binding site (red) of let-7b in the chicken *IGF2BP3* mRNA 3′UTR is highly conserved among vertebrates. **(C)** Dual-luciferase reporter assay indicated that let-7b can bind to the predicted binding site in the chicken *IGF2BP3* mRNA 3′UTR. Data were displayed as normalized fold change in relative luciferase activity (Firefly luciferase/Renilla luciferase, relative value of Mock group was set as 1). The data are mean ± S.E.M. with three cultures per group, and three wells per culture were assayed (*n* = 9/treatment group). Independent sample *t*-test was used to analyze the statistical differences between groups. ^**^p < 0.01.

Our previous microarray data showed that let-7b expression is significantly higher in the skeletal muscle of 7 week (w) dwarf chickens than in 7 w normal chickens (Figure 1A), and IGF2BP3 mRNA expression is significantly lower in the skeletal muscle of 7 week (w) dwarf chickens than in 7 w normal chickens (Figure 2A). The following qPCR results are consistent with the microarray data (Figures 1C,D). In addition, the Western blot analysis also showed that the expression of IGF2BP3 protein in dwarf chicken was significantly lower compared to that in normal chicken (Figures 1E,F). Therefore, let-7b and IGF2BP3 showed opposite expression trends in the skeletal muscle of dwarf and normal chickens.

### *IGF2BP3* is a target gene of let-7b

To verify whether *IGF2BP3* is a target gene of let-7b, we used TargetScan (http://www.targetscan.org) and miRDB (http://mirdb.org/index.html) to predict the target relationship between let-7b and *IGF2BP3*. Both of these two miRNA target prediction software showed that the 3′UTR of chicken *IGF2BP3* mRNA has a potential binding site of let-7b (Figure 2A), and this binding site is conserved among vertebrates (Figure 2B). Next, we used dual-luciferase reporter gene assay to validate the binding ability of let-7b to the potential binding site. Results showed that the overexpression of let-7b significantly repressed the relative luciferase activity of the cells transfected with wild-type IGF2BP3-3′UTR reporter, and mutation of the predicted binding site would abolish the inhibition effect of let-7b to the reporter (Figure 2C). Collectively, these results indicate that *IGF2BP3* is a direct target gene of let-7b in chicken.

### Let-7b has an enhanced inhibitory effect on *IGF2BP3* expression in dwarf myoblast than in normal myoblast

**Figure 3:**
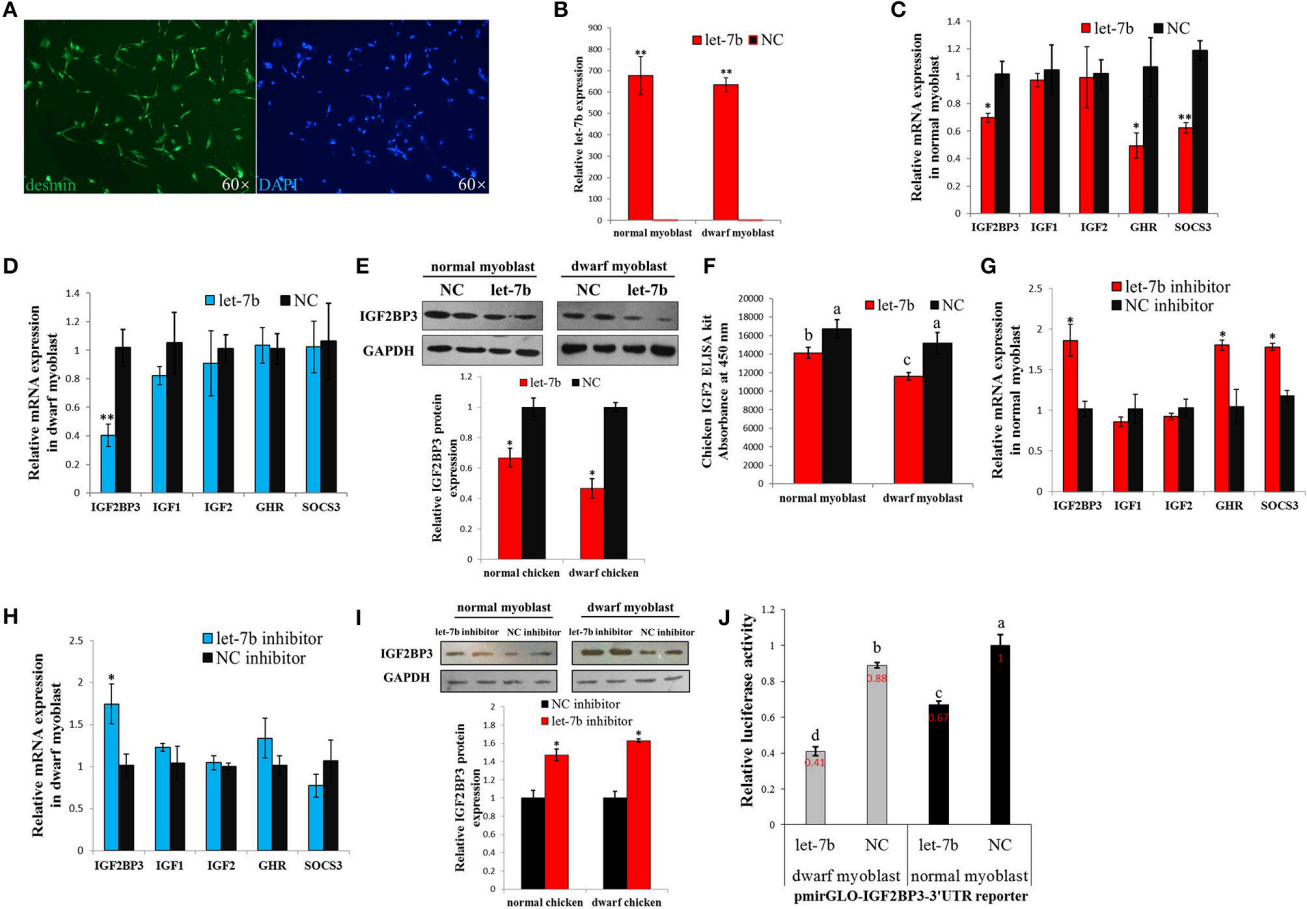
Let-7b has an enhanced inhibitory effect on *IGF2BP3* expression in dwarf myoblast than in normal myoblast. **(A)** Desmin immunostaining of primary myoblast. **(B)** Relative let-7b expression in normal and dwarf myoblasts after transfection of let-7b mimic or NC mimic. **(C)** Relative mRNA expression after transfection of let-7b mimic or NC mimic in myoblast isolated from normal chicken. **(D)** Relative mRNA expression after transfection of let-7b mimic or NC mimic in myoblast isolated from dwarf chicken. **(E)** Relative IGF2BP3 protein expression in normal and dwarf myoblast after transfection of let-7b mimic or NC mimic. **(F)** ELISA analyzes of IGF2 protein expression in normal and dwarf myoblast after transfection of let-7b mimic or NC mimic. **(G)** Relative mRNA expression after transfection of let-7b inhibitor or NC inhibitor in myoblast isolated from normal chicken. **(H)** Relative mRNA expression after transfection of let-7b inhibitor or NC inhibitor in myoblast isolated from dwarf chicken. **(I)** Relative IGF2BP3 protein expression in normal and dwarf myoblast after transfection of let-7b inhibitor or NC inhibitor. **(J)** Relative luciferase activity of dwarf and normal myoblasts transfected with let-7b mimics and pmirGLO-IGF2BP3-3′UTR. Data were displayed as normalized fold change in relative luciferase activity (Firefly luciferase/Renilla luciferase, relative value of NC group in normal myoblast was set as 1). The data in **(B–D,F–H,J)** are mean ± S.E.M. with three cultures per group, and three wells per culture were assayed (*n* = 9/treatment group). The data in **(E,I)** are mean ± S.E.M. from three independent experiments done in duplicate (*n* = 6/treatment group). For **(B–I)**, independent sample *t*-test was used to analyze the statistical differences between groups. ^*^p < 0.05; ^**^p < 0.01. For **(F,J)**, different letters above the bars indicate significant differences (*p* < 0.05) by using the Duncan's Multiple Range Test, at the *p* < 0.05 significance level.

To further study the regulation of let-7b on *IGF2BP3* in dwarf and normal chicken skeletal muscle, we transfected let-7b mimic to the primary myoblast (Figure 3A) of dwarf and normal chickens, respectively (Figure 3B). In normal myoblast, let-7b overexpression significantly reduced *IGF2BP3* expression by about 30%, and the expression of *GHR* gene, which is another let-7b target gene, is also down-regulated (Figure 3C). However, the mRNA expressions of *IGF1* and *IGF2*, which are IGF2BP3 downstream genes, have no change (Figure 3C). In dwarf myoblast, let-7b overexpression significantly reduced the relative expression of *IGF2BP3* by more than 60%, and the expression of *GHR, IGF1* and *IGF2* have no change (Figure 3D). Immunoblotting results also showed that let-7b overexpression reduced the relative IGF2BP3 protein expression by about 35% in normal chicken, whereas this reduction was more than 50% in dwarf chicken (Figure 3E). Additionally, IGF2 protein expression was down-regulated both in dwarf and in normal myoblast after let-7b overexpression (Figure 3F), and the IGF2 protein level was lower in dwarf myoblast than in normal myoblast after let-7b transfection (Figure 3F). On the other hand, let-7b inhibition significantly increased *IGF2BP3, GHR* and *Suppressor of Cytokine Signaling 3* (*SOCS3)* mRNA expression in normal myoblast (Figure 3G), whereas its inhibition in dwarf myoblast can only increase *IGF2BP3* mRNA expression (Figure 3H). The inhibition of let-7b also increased IGF2BP3 protein level in both normal and dwarf myoblast (Figure 3I).

To further understand whether there is any difference in the binding of let-7b to *IGF2BP3* between dwarf and normal myoblasts, we overexpressed let-7b and NC mimics, respectively, to the myoblasts transfected with pmirGLO-IGF2BP3-3′UTR reporter. In both dwarf and normal myoblasts, let-7b overexpression significantly inhibited luciferase activity of the reporters (Figure 3J). With let-7b overexpression, the relative luciferase activity was significantly lower in dwarf myoblast than in normal myoblast (0.41 vs. 0.67). Without let-7b overexpression, the relative luciferase activity was also lower in dwarf myoblast than in normal myoblast (0.88 vs. 1). Therefore, these results suggested that let-7b inhibits IGF2BP3 expression in chicken myoblast, and the binding activity of let-7b to the 3′UTR of *IGF2BP3* mRNA is significantly higher in dwarf myoblast than in normal myoblast.

### Let-7b inhibits chicken primary myoblast proliferation through represses its target gene *IGF2BP3*

**Figure 4:**
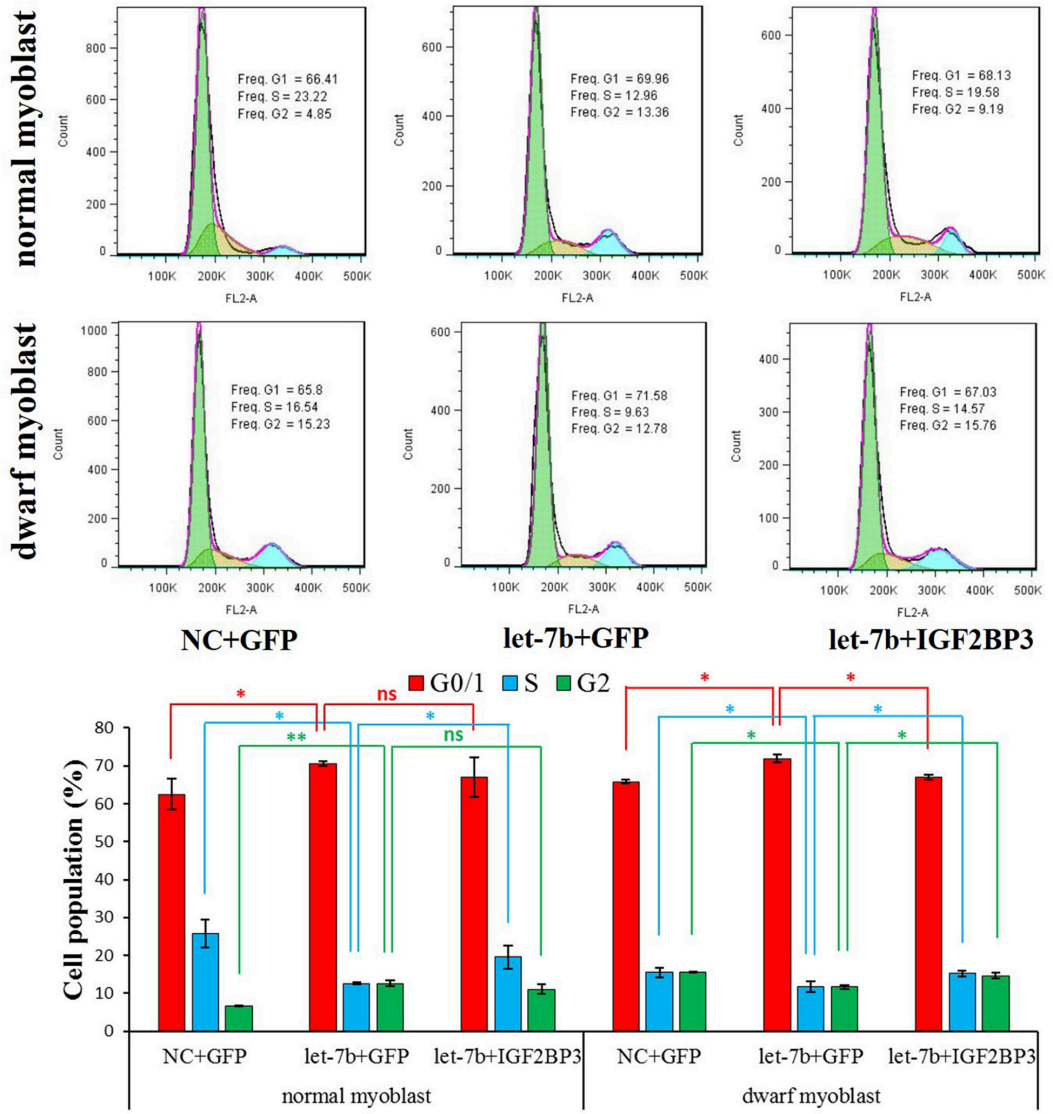
Let-7b inhibits chicken primary myoblast proliferation through represses its target gene *IGF2BP3*. Cell cycle analysis of normal and dwarf myoblasts 36 h after transfection. Propidium iodide staining for DNA content and FACS was used to determine the percentage of cells in G1, S, and G2. pcDNA3.1-GFP was used as a control vector. The “NC+GFP” group and the “let-7b+IGF2BP3” group were compared with the “let-7b+GFP” group, respectively. Results are shown as the mean ± S.E.M with three cultures per group, and three wells per culture were assayed (*n* = 9/treatment group). Independent sample *t*-test was used to analyze the statistical differences between groups. ^*^p < 0.05; ^**^p < 0.01.

To test whether let-7b can regulate chicken myoblast proliferation or not, we next detect the effect of let-7b on the regulation of myoblast cell cycle. In both normal and dwarf myoblasts, let-7b overexpression significantly reduce the number of cells that progressed to S phase, and the number of cells that progressed to G0/1 phase was significantly increased (Figures 4A,B). Interestingly, when we co-transfected let-7b and IGF2BP3 overexpression vector into dwarf myoblast, the significant changes of the number of S, G0/1, and G2 phase cells induced by let-7b were rescued (Figures 4A,B). However, the co-transfection of let-7b and *IGF2BP3* cannot fully rescue the inhibition effect of let-7b in cell cycle of normal myoblast. Only the number of cells that progressed to S phase was partially rescued (Figures 4A,B). Therefore, these results indicated that let-7b inhibits chicken primary myoblast proliferation at least in part through represses its target gene *IGF2BP3*.

## Discussion

The GH/GHR/IGFs somatotropic axis plays a key role in the regulation of metabolism and physiological processes (Renaville et al., 2002). GH activates GHR by dimerizing two identical receptor subunits, leading to Janus family of protein tyrosine kinases 2 kinase activation that in turn activates a number of intracellular pathways (Brown et al., 2005). Additionally, GH exerts many of its actions through IGFs, which is required for normal embryonic development and postnatal growth in vertebrates (Powell-Braxton et al., 1993). In our previous work, we found let-7b is able to regulate the GH/GHR/IGFs somatotropic axis through inhibiting *GHR* gene expression (Lin et al., 2012). Here, with a further study of let-7b, we found another role of let-7b in the regulation of this somatotropic axis. IGF2BP3, an *IGF2* mRNA binding protein that can regulate IGF2 expression and function (Liao et al., 2005), is also a direct target gene of let-7b. Therefore, these works suggesting an important regulatory role of let-7b in the GH/GHR/IGFs somatotropic axis.

The let-7 family is one of the first identified miRNAs and known to be differentially expressed between embryo and mature tissues (Yanaihara et al., 2006). Let-7b, a member of the let-7 family, is characterized to be highly conserve, tissue specific, and has important roles in regulating cell development (Pasquinelli et al., 2000; Gao et al., 2014). However, the role of let-7b in skeletal muscle development still remains unknown. In our previous microarray analysis, we found that let-7b is differentially expressed between the skeletal muscles of dwarf and normal chicken, suggesting that it is potentially involved in the regulation of chicken muscle development (Lin et al., 2012; Luo et al., 2016). Here, we validated that *IGF2BP3* is a conserve and direct target gene of let-7b, and the inhibition of *IGF2BP3* by let-7b would result in cell cycle arrest of chicken primary myoblast, demonstrating that let-7b can regulate myoblast proliferation. Additionally, *GHR* is another target gene of let-7b in chicken. GHR is essential for the activation of cell proliferation (Dinerstein et al., 1995; Conway-Campbell et al., 2008). GHR-deficient mice exhibited reduced myofiber diameter and myoblast fusion that is independent of the function of IGF1 (Sotiropoulos et al., 2006; Schuenke et al., 2008). Therefore, the inhibition of *GHR* and *IGF2BP3* by let-7b in chicken myoblast will not only result in cell cycle arrest, but also may regulate the other developmental processes of myoblast.

In this study, we found that the protein and mRNA levels of *IGF2BP3* were significantly down-regulated in skeletal muscle of dwarf chicken at 7 w of age. Furthermore, from let-7b overexpressed analysis, we observed that let-7b significantly reduced mRNA and protein levels of *IGF2BP3* without affecting *IGF2* mRNA levels, but the IGF2 protein was significantly reduced in both normal and dwarf myoblasts, suggesting that let-7b-mediated down-regulation of *IGF2BP3* could further reduce IGF2 expression at the post-transcriptional level. These results are consistent with the previous study that IGF2BP3 functions as a translational activator for IGF2 (Liao et al., 2005). IGF2BP3 knocked-down can significantly decrease the expression levels of intracellular and secreted IGF2 (Liao et al., 2005). Additionally, IGF2 is an important growth factor that can promote cell development and growth. It can bind to two types of cell surface receptors known as IGF1 receptor and IGF2 receptor, stimulate cell proliferation and inhibit apoptosis (Bergman et al., 2013). The reduced expression of *IGF2BP3* in dwarf chicken would result in the inhibition of IGF2 and cell growth. Therefore, these results indicated that the increased expression of let-7b in dwarf chicken results in *IGF2BP3* repression, which further inhibits IGF2 translation and cell proliferation through let-7b-IGF2BP3-IGF2 pathway. This pathway may be one of the reason for the loss of muscle mass in dwarf chicken.

**Figure 5:**
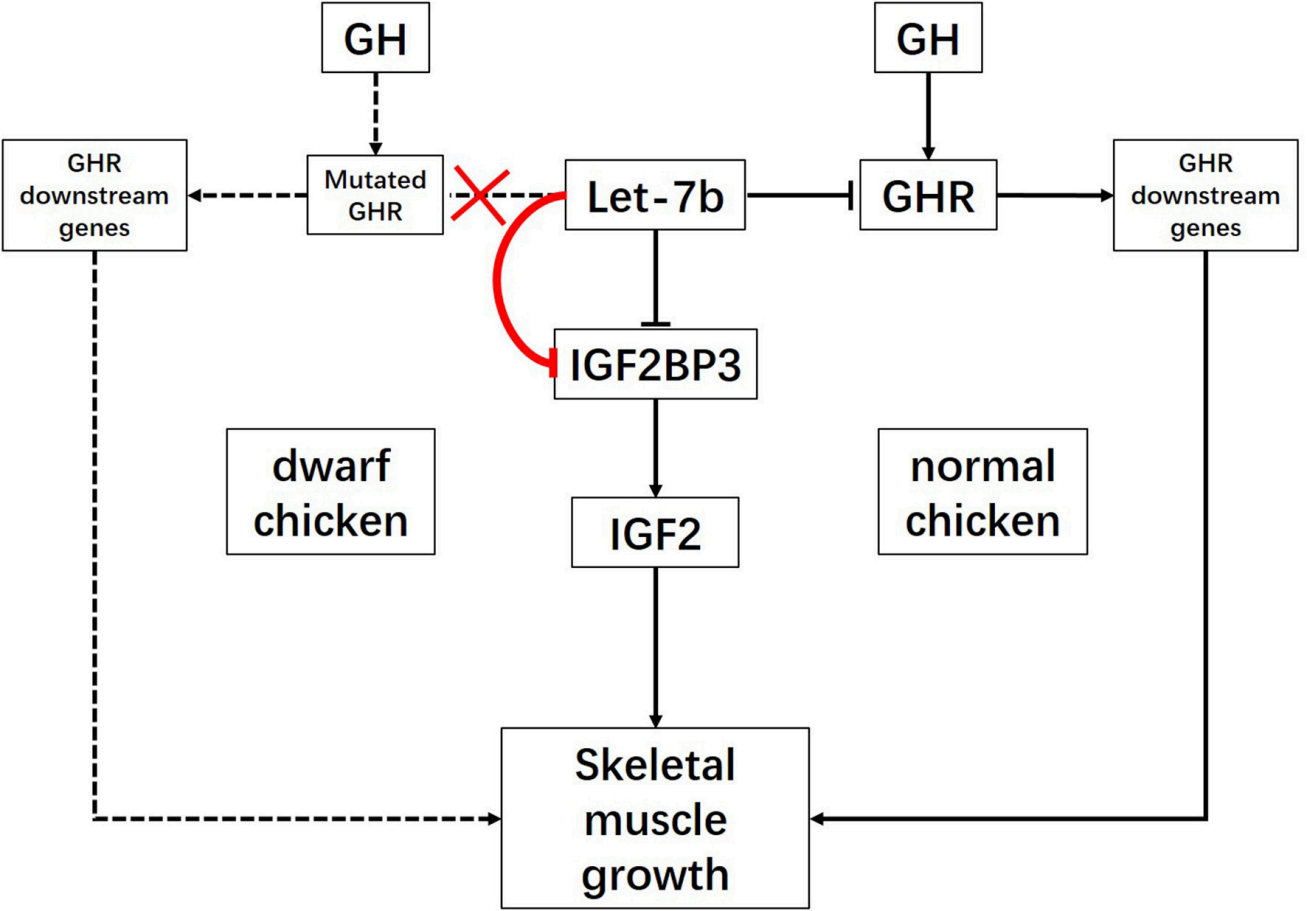
Schematic illustration for signaling pathways of skeletal muscle growth regulated by let-7b. let-7b can inhibit skeletal muscle development through let-7b-IGF2BP3-IGF2 signaling pathway and let-7b-GHR-GHR downstream genes pathway in normal chicken. However, the large deletion mutation at the exon 10 and 3′UTR of *GHR* gene in dwarf chicken lead to disruption of let-7b binding site in *GHR* 3′UTR. In this case, let-7b would enhance its inhibition on *IGF2BP3* expression. Therefore, let-7b inhibits skeletal muscle development only through let-7b-IGF2BP3-IGF2 signaling pathway with enhancing effect in dwarf chicken.

Combined previous studies with our present results, we can establish gene regulatory network for let-7b mediated skeletal muscle development (Figure 5). In this network, we concluded that the let-7b mediated regulatory pathway is different between dwarf chicken and normal chicken. For normal chicken, let-7b not only can promote skeletal muscle growth through let-7b-GHR-GHR downstream genes signaling pathway, but also can inhibit muscle growth via let-7b-IGF2BP3-IGF2 signaling pathway. For the dwarf chicken, as the deletion mutation not only results in dysfunction of GHR and dwarf phenotype of chicken, but also leads to the loss of the ability of let-7b to pair with sequences in *GHR* mRNA 3′UTR (Lin et al., 2012), the regulation of *GHR* gene was affected (Figure 5). On the one hand, the higher expression of let-7b in dwarf chicken results in the more significant decrease of IGF2BP3 in dwarf chicken skeletal muscle and leads to decrease of IGF2 expression, which finally repressed skeletal muscle growth through let-7b-IGF2BP3-IGF2 signaling pathway. On the other hand, the loss of target binding site of let-7b in dwarf *GHR* mRNA might result in increasing binding of let-7b to *IGF2BP3*, because the same amount of let-7b expression in dwarf and normal myoblast leads to different IGF2BP3 repression. Both the relative expression levels of *IGF2BP3* mRNA and protein were reduced more in dwarf myoblast than in normal myoblast, and the binding activity of let-7b in dwarf myoblast is significantly higher than that in normal myoblast. Previous studies have shown that polymorphism in target site and synaptic stimulation would modify miRNA binding to its target site (Schratt et al., 2006; Wu et al., 2013; Bhaumik et al., 2014), but we have not observed that the loss of one miRNA target site would increase its binding to another target site before. This phenomenon is interesting and needed for further study. Collectively, let-7b regulates skeletal muscle growth through two signaling pathways in normal chicken, but only one signaling pathway with enhancing effect in dwarf chicken.

The dwarf chicken study here is caused by a recessive mutation of the *GHR* gene (Duriez et al., 1993; Huang et al., 1993; Tanaka et al., 1995; Hull et al., 1999). This mutation results in shorter shanks, higher serum GH, lower serum IGF1 and IGF2, and many other phenotypic and physiological alterations in dwarf chicken (Scanes et al., 1989; Burnside et al., 1992; Ouyang et al., 2012). The average body weight, skeletal muscle fiber diameter and the number of muscle fiber were reduced in homozygous (*dwdw*) dwarf chickens (Knizetova, 1993; Luo et al., 2016). Although the root cause of the formation of the dwarf chicken has been studied clearly, the precise mechanism and pathways result in the loss of skeletal muscle in dwarf chicken still remain unclear. In this study, we found that let-7b mediated pathway is one of the reasons that can lead to inhibition of skeletal muscle growth in dwarf chickens. The higher expression of let-7b might be a feedback result of the loss function of *GHR* gene in dwarf chicken. *GHR* gene defection would lead to the decrease of IGF1, which is able to negatively alter let-7 family expression (Martin et al., 2012). Additionally, growth factors can also suppress let-7 expression through MAPK signaling pathway (Dangi-Garimella et al., 2009), which is inactivated in dwarf chicken (Luo et al., 2016). Therefore, the up-regulation of let-7b in dwarf chicken might be due to the loss-of-function of *GHR* and down-regulation of *IGF1*. Let-7b up-regulation balance the inhibiting effect of let-7b on cell proliferation through enhancing its binding to *IGF2BP3*.

## Author contributions

SL, WL, QN, YL, and XZ designed the experiments, SL, WL, YY, EB, and XZ wrote the manuscript. SL, WL, and YY did the experiments.

### Conflict of interest statement

The authors declare that the research was conducted in the absence of any commercial or financial relationships that could be construed as a potential conflict of interest.

## Abbreviations

miRNA: microRNA
GH: growth hormone
GHR: growth hormone receptor
IGF: Insulin-like growth factor
IGF2BP3: Insulin-like growth factor 2 binding protein 3
IGFBP: insulin like growth factor mRNA binding protein
UTR: untranslated region
qPCR: quantitative polymerase chain reaction
NC: negative control
GM: growth medium
DF-1: chicken embryo fibroblast cell line
HRP: horseradish peroxidase
ELISA: enzyme-linked immunosorbent assay
SOCS3: suppressor of cytokine signaling 3
w: week.
